# Using Motor Learning to Predict Cognitive and Functional Decline in Mild Cognitive Impairment and Alzheimer’s Disease

**DOI:** 10.1002/alz.090898

**Published:** 2025-01-03

**Authors:** Vincent Koppelmans, Kevin Duff, Sydney Y Schaefer

**Affiliations:** ^1^ University of Utah, Salt Lake City, UT USA; ^2^ NIA‐Layton Aging & Alzheimer’s Disease Research Center, Portland, OR USA; ^3^ Arizona State University, Tempe, AZ USA

## Abstract

**Background:**

Predicting decline over the course of Mild Cognitive Impairment (MCI) and Alzheimer’s Disease (AD), especially on relatively short time frames, is vital for appropriate treatment planning and to tailor patient and support systems’ expectations. The current study tested if a functional upper limb motor learning task could predict one‐year change in cognition and daily function.

**Method:**

Cognitively unimpaired (n = 61), MCI (n = 35), and AD (32) older subjects (age: 74.6 ±5.7 years) completed a simulated feeding task (SFT) at baseline for which they used a plastic spoon to scoop two raw kidney beans at a time with their non‐dominant hand from a central cup and transport them to one of three distal cups. They completed this sequence five times for a total of 15 out‐and‐back movements. Time was recorded as outcome measure, and 6 trials of the test were completed. This test was completed 6 times with trial time as the outcome measure for each trial. At baseline and one‐year follow‐up visits, the Mini Mental Status Examination (MMSE) was administered to assess cognition and a knowledgeable collateral completed the Quick Dementia Rating System (QDRS) to assess daily functioning. Linear regression analysis, adjusted for age, sex, and years of education, was used to test the association between SFT performance over the six trials (mean/sd) and regression‐based one‐year change scores on the QDRS and MMSE.

**Result:**

SFT performance speed and variability significantly predicted change in performance on the QDRS (total score, cognitive domain score, and behavioral domain score; p = .013‐.024). SFT variability (a measure of learning), but not average performance, over six trials predicted change on the MMSE (p = .031). These effects were small to medium in size (eta^2 = 0.04‐0.05).

**Conclusion:**

Speed and variability on a brief, easy‐to‐administer (∼5‐7 minutes), cost‐efficient functional upper limb motor learning task predicted cognitive and functional change over one year across the AD spectrum. These promising results suggest that the SFT could be used in clinical practice to help predict disease progression, as well as a screening tool in clinical trials in MCI and AD.

